# Anticancer and Antibiotic Rhenium Tri- and Dicarbonyl Complexes: Current Research and Future Perspectives

**DOI:** 10.3390/molecules27020539

**Published:** 2022-01-15

**Authors:** Kevin Schindler, Fabio Zobi

**Affiliations:** Department of Chemistry, Fribourg University, Chemin du Musée 9, 1700 Fribourg, Switzerland; kevin.schindler@unifr.ch

**Keywords:** rhenium, dicarbonyl, tricarbonyl, anticancer, antibiotic, homonuclear, heteronuclear

## Abstract

Organometallic compounds are increasingly recognized as promising anticancer and antibiotic drug candidates. Among the transition metal ions investigated for these purposes, rhenium occupies a special role. Its tri- and dicarbonyl complexes, in particular, attract continuous attention due to their relative ease of preparation, stability and unique photophysical and luminescent properties that allow the combination of diagnostic and therapeutic purposes, thereby permitting, e.g., molecules to be tracked within cells. In this review, we discuss the anticancer and antibiotic properties of rhenium tri- and dicarbonyl complexes described in the last seven years, mainly in terms of their structural variations and in vitro efficacy. Given the abundant literature available, the focus is initially directed on tricarbonyl complexes of rhenium. Dicarbonyl species of the metal ion, which are slowly gaining momentum, are discussed in the second part in terms of future perspective for the possible developments in the field.

## 1. Introduction

The demand for anticancer and antibiotic drugs is increasing worldwide since the current pipelines remain insufficient against the increasing emergence and spread of resistance. The cancer burden worldwide remains among the leading causes of death in the world, in particular in high-income countries [1]. Concurrently, antimicrobial resistance and invasive fungal infections are major emerging threats to public health, causing serious issues in the successful prevention and treatment of persistent diseases [2]. Chemotherapy, which involves the administration of cytotoxic chemical compounds, represents the principal form of cancer treatment, alongside strategies such as surgical removal and radiotherapy. The administration of platinum-based complexes in chemotherapy results in severe side effects [3]. Additionally, many cancer types display acquired resistance to platinum-based agents, further limiting their use in clinical practice. The shortcomings of these complexes have encouraged the pursuit of novel transition metal anticancer agents that bypass the side effects associated with platinum agents while maintaining superior anticancer activity. Indeed, various transition-metal-based anticancer compounds (including copper, gallium, ruthenium, palladium, rhenium, gold and titanium) have been developed [4]. However, none of them has so far been approved for clinical anticancer treatment. We believe that the discovery and development of a highly active and selective anticancer complexes will have a remarkable impact in the field and would encourage industries to invest in organometallic therapy research. Undeniably, unlike organic molecules, metal complexes can adopt unique 3D structures (e.g., octahedral, square pyramidal, trigonal bipyramidal structures), and offer the possibility to create a wide variety of anticancer, antiparasitic, and antimicrobial drugs with unique modes of action unavailable for organic drugs. 

Of the different metal ions being investigated, complexes of rhenium are among the most promising candidates for therapeutic applications [5,6,7,8,9,10,11,12,13]. Compared to platinum, e.g., rhenium is more economically viable, and its tricarbonyl compounds possess luminescent properties that permit theragnostic utilizations [8]. Additionally, the thermodynamic stable product of the aerobic decomposition of many rhenium species is perrhenate (ReO_4_^-^), whose salts of potassium and sodium are as toxic as sodium chloride [14]. This makes perrhenate one of the least toxic transition metal oxides, and rhenium species are obviously attractive for anticancer and antibiotic applications in terms of possibly reducing side-effects associated with its chemotherapies. Furthermore, rhenium compounds are already used in clinical therapies. Different Re radioisotopes, mainly ^186^Re and ^188^Re, are used in the treatment of, e.g., inflammatory joint damage [15,16], bone metastases [17], malignant tumors, and rheumatoid arthritis [18]. 

In this review, we focus on the anticancer and antibiotic properties of rhenium carbonyl complexes, mainly in terms of their structural variations and efficacy. We have selected only the latest studies (published in the last seven years) since the important publication of Gasser’s review in 2014 [5]. The fields have been thriving in the last decade, and several excellent reviews have analyzed rhenium complexes [6,7,8,9,10,11,12,13] in terms, e.g., of radiopharmaceutical developments [6], therapeutic and diagnostic applications [7,8,9,10], mechanism of action [11], photodynamic therapy [12], or nanoparticles functionalization [13]. With a few exceptions, here we discuss only “active” complexes, defined as such by in vitro IC_50_ and MIC values ≤ 5 μM (anticancer compounds) and <10 µg/mL (antibiotic compounds), respectively. It will become immediately apparent to the reader that tricarbonyl complexes of rhenium dominate the research fields, but dicarbonyl complexes are slowly gaining momentum. These latter species are discussed in terms of future perspective for the possible developments in these important investigations. 

## 2. Anticancer Complexes

### 2.1. Mononuclear Complexes

**Figure 1:**
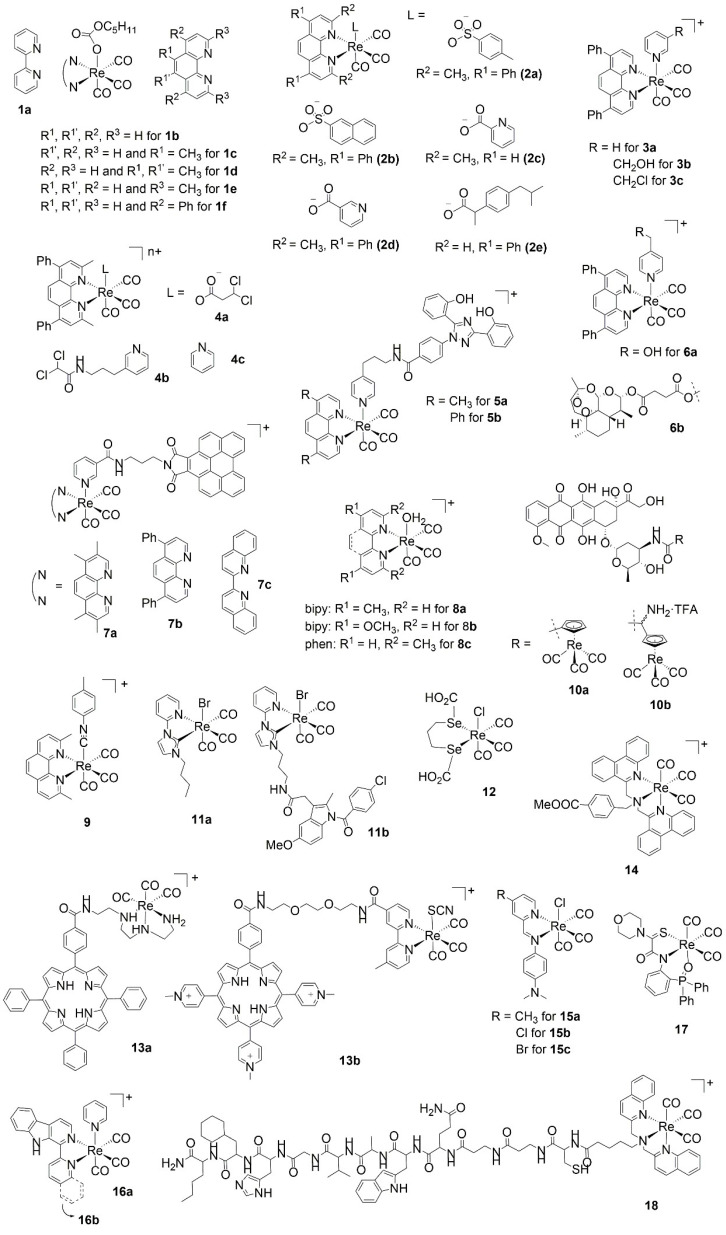
Selected structures of anticancer rhenium tricarbonyl complexes.

The overwhelming majority of rhenium carbonyl complexes tested in the last seven years are those of the *fac*-[Re(CO)_3_]^+^ core. A review of the literature revealed that the most active species (in vitro IC_50_ values ≤ 5 μM) are those bound to a diimine bidentate ligand in the form of derivatized 2,2’-bipyridine (bipy) and 1,10-phenanthroline (phen, Figure 1 and Table 1). Of the two ligand systems, phen derivatives dominate, with 82% of the total of active complexes; lipophilic and neutral compounds are in general more potent. The proportion is substantially skewed towards neutral lipophilic phenanthroline complexes due to the work of Banerjee and co-authors [19] and the subsequent study of Weber, Mandal, and co-authors [20]. The groups reported, respectively, the anticancer properties of pentylcarbanato (**1a/f**, Figure 1 and Table 1) and sulfonate or carboxylato (**2a/e**) organorhenium compounds of phen derivatives against different cell lines (nearly 100 molecules in total). The pentylcarbanato complexes **1a**–**1f** are less nephrotoxic than cisplatin. They have significant cytotoxicity against the MDA-MB-468 (HTB-132) triple-node-negative breast cancer cell line, with IC_50_ values below 5 μM and may thus potentially find applications in the treatment of this highly malignant type of tumor [19]. Sulfonate or carboxylate compounds **2a/e** are active against both hormone-dependent MCF-7 and hormone-independent triple negative MDA-MB-231 breast cancer cells, with several of them far more potent than the conventional drug cisplatin. Indeed, of the 88 compounds tested, 57 showed IC_50_ values below 5 μM (several of which below 2 μM); 6 molecules showed IC_50_ values below 1 μM; and 1 complex, the 2,9-dimethyl-1,10-phenanthroline picolinato complex **2c**, showed an IC_50_ value against MCF-10A cells of 0.023 µM. DNA-binding and structure–activity relationship (SAR) studies suggest that the anticancer activity of the species increases with the increasing lipophilicity, it being roughly consistent with the DNA-binding (including DNA partial intercalation) of the same [20]. 

**Table 1:** IC_50_ values of selected anticancer rhenium complexes toward human cancer cell lines.

Compound	IC_50_ (µM)	IC_50_ (µM) of Ref. Drug ^1^	Cell Line	Ref.
**1a**	3 ± 2.5	5 ± 2.5	HTB-132	[19]
**1a**	5 ± 2.5	1 ± 2.5	Balb/c	[19]
**1b**	2 ± 1.5	5 ± 2.5	HTB-132	[19]
**1b**	5 ± 3.5	1 ± 2.5	Balb/c	[19]
**1c**	3 ± 2.5	5 ± 2.5	HTB-132	[19]
**1c**	4 ± 2.5	1 ± 2.5	Balb/c	[19]
**1d**	3 ± 4.5	5 ± 2.5	HTB-132	[19]
**1d**	4 ± 2.5	1 ± 2.5	Balb/c	[19]
**1e**	2 ± 2.5	5 ± 2.5	HTB-132	[19]
**1e**	5 ± 5.5	1 ± 2.5	Balb/c	[19]
**1f**	4 ± 6.5	5 ± 2.5	HTB-132	[19]
**1f**	5 ± 1.5	1 ± 2.5	Balb/c	[19]
**2a**	0.34 ± 0.30	n.i.	MCF-7A	[20]
**2a**	0.25 ± 0.34	n.i.	MDA-MB-231	[20]
**2b**	0.85 ± 0.26	n.i.	MCF-7A	[20]
**2b**	0.72 ± 0.24	n.i.	MDA-MB-231	[20]
**2c**	0.43 ± 0.23	n.i.	MCF-7A	[20]
**2c**	0.02 ± 0.002	n.i.	MCF-10A	[20]
**2d**	0.59 ± 0.15	n.i.	MDA-MB-231	[20]
**2e**	0.96 ± 0.26	n.i.	MCF-7A	[20]
**3a**	3.9 ± 0.7	21.5 ± 2.5	A549	[21]
**3a**	1.2 ± 0.5	65.6 ± 1.6	A549R	[21]
**3a**	0.95 ± 0.11	8.9 ± 1.0	HeLa	[21]
**3a**	3.1 ± 0.5	29.9 ±2.1	LO2	[21]
**3b**	2.7 ± 0.5	65.6 ± 1.6	A549R	[21]
**3b**	1.7 ± 0.4	8.9 ± 1.0	HeLa	[21]
**3c**	3.4 ± 0.6	21.5 ± 2.5	A549	[21]
**3c**	0.75 ± 0.12	65.6 ± 1.6	A549R	[21]
**3c**	0.52 ± 0.07	8.9 ± 1.0	HeLa	[21]
**4a**	3.7 ± 0.2	>50	NCI-H1229	[22]
**4b**	0.8 ± 0.1	>50	NCI-H1229	[22]
**4b**	2.2 ± 0.2	>50	RKO	[22]
**4b**	4.0 ± 1.2	>50	MCF-7	[22]
**4b**	4.1 ± 0.9	>50	A549	[22]
**4b**	4.3 ± 0.7	>50	A549R	[22]
**4c**	2.9 ± 0.1	>50	NCI-H1229	[22]
**5a**	0.7 ± 0.8	40.5 ± 2.2	MDA-MB-231	[23]
**5b**	0.4 ± 0.1	40.5 ± 2.2	MDA-MB-231	[23]
**6a**	4.8 ± 0.2	20.0 ± 2.1	HeLa	[24]
**6a**	5.1 ± 0.3	18.2 ± 1.5	A549	[24]
**6b**	0.8 ± 0.1	20.0 ± 2.1	HeLa	[24]
**6b**	1.1 ± 0.2	18.2 ± 1.5	A549	[24]
**6b**	1.5 ± 0.2	86.5 ± 9.0	A549R	[24]
**6b**	1.0 ± 0.1	22.4 ± 2.0	HepG2	[24]
7a *	0.27 ± 0.02	n.i.	HeLa	[25]
7b *	2.21 ± 0.12	n.i.	HeLa	[25]
7c *	1.51 ± 0.01	n.i.	HeLa	[25]
**8a**	4.3 ± 1.6	1.0 ± 0.3	KB-3-1	[26]
**8a**	3.5 ± 2.8	0.23 ± 0.07	A2780	[26]
**8a**	4.7 ± 1.4	8.2 ± 1.8	A2780CP70	[26]
**8a**	3.9 ± 4.6	12.4 ± 8.5	A549 CisR	[26]
**8b**	0.77 ± 0.17	1.0 ± 0.3	KB-3-1	[26]
**8b**	2.2 ± 1.8	0.23 ± 0.07	A2780	[26]
**8b**	2.8 ± 2.5	8.2 ± 1.8	A2780CP70	[26]
**8c**	0.92 ± 0.20	1.0 ± 0.3	KB-3-1	[26]
**8c**	2.2 ± 0.2	0.23 ± 0.07	A2780	[26]
**8c**	3.0 ± 0.7	8.2 ± 1.8	A2780CP70	[26]
**8c**	4.5 ± 0.7	0.75 ± 0.43	H460	[26]
**8c**	4.1 ± 0.9	0.43 ± 0.14	MRC-5	[26]
**9**	1.7 ± 0.7	1.3 ± 0.1	A2780	[27]
**9**	1.9 ± 1	12 ± 3	A2780CP70	[27]
**9**	1.4 ± 0.2	6.6 ± 0.7	HeLa	[27]
**9**	1.4 ± 0.6	5.6 ± 0.5	A549	[27]
**9**	1.9 ± 0.2	1.7 ± 0.2	HEK293	[27]
**10a**	0.34 ± 0.03	0.11 ± 0.02 doxorubicin	HeLa	[28]
**10b**	1.65 ± 0.26	0.11 ± 0.02 doxorubicin	HeLa	[28]
**11a**	4.0 ± 1.2	6.8 ± 2.0	ASPC1	[29]
**11b**	4.8 ± 0.8	8.7 ± 4.3	HPAF-II	[29]
**12**	5	n.i.	MDA-MB231	[8]
13a *	0.9 ± 0.1	n.i.	HeLa	[30]
13b *	3.3 ± 2.3	n.i.	HeLa	[30]
**14**	1 − 2.5	n.i.	BJAB	[31]
**15a**	5.1 ± 0.5	1.1 ± 0.4	A2780	[32]
**15a**	3.7 ± 0.6	14.3 ± 1.3	A2780CP70	[32]
**15b**	4.3 ± 1.3	1.1 ± 0.4	A2780	[32]
**15b**	4.1 ± 1.7	14.3 ± 1.3	A2780CP70	[32]
**15c**	3.2 ± 0.3	1.1 ± 0.4	A2780	[32]
**15c**	3.6 ± 0.2	14.3 ± 1.3	A2780CP70	[32]
**16a**	4.3 ± 0.4	8.2 ± 0.7	A549	[33]
**16a**	3.0 ± 0.2	41.5 ± 5.2	A549R	[33]
**16a**	2.2 ± 0.3	7.7 ± 0.8	HeLa	[33]
**16a**	2.4 ± 0.4	9.4 ± 1.0	MCF-7	[33]
**16b**	2.2 ± 0.2	8.2 ± 0.7	A549	[33]
**16b**	2.1 ± 0.1	41.5 ± 5.2	A549R	[33]
**16b**	1.8 ± 0.2	7.7 ± 0.8	HeLa	[33]
**16b**	2.2 ± 0.3	9.4 ± 1.0	MCF-7	[33]
**17**	4.5 ± 0.5	18.0 ± 2.0	HCT116	[34]
18 *	5.3 ± 1.0	n.i.	HeLa	[35]
**19**	1.1 ± 0.1	10.7 ± 0.9	U2SO	[36]
**19**	1.8 ± 0.1	9.2 ± 0.1	HeLa	[36]
**19**	0.8 ± 0.1	9.6 ± 0.8	A549	[36]
**20**	8.61	n.i.	MDA-MB-231	[37]
21 *	1.4 ± 1.3	n.i.	HeLa	[38]
21 *	0.5 ± 0.2	n.i.	H460M2	[38]
21 *	0.5 ± 0.1	n.i.	HBL-100	[38]
**22**	6.38 ± 1.18	37.30 ± 5.69	A2780	[39]
**23**	7.4 ± 1.5	13 ± 1.8	MDA-MB-231	[40]
**23**	7.8 ± 3.3	14 ± 1.0	HCT-116	[40]
**26**	4.5 ± 0.1	0.7 ± 0.2	C6	[41]
**27**	1.7 ± 0.3	20.4 ± 3.4	MDA-MB-231	[42]
**27**	1.1 ± 0.4	14 ± 3.5	MCF-7	[42]
**27**	1.3 ± 0.2	1.0 ± 0.2	A2780	[42]
**27**	3.3 ± 0.3	2.9 ± 0.8	MCF-10A	[42]
28 *	13.5 ± 4.1	n.i.	HeLa	[43]
29 *	4.8 ± 1.3	n.i.	A2780CP70	[44]

^1^ Cisplatin if not indicated; n.i. stands for “not indicated”; * refers to photoactivated compounds.

Other prominent examples of highly active lipophilic, but cationic, phenanthroline complexes have been described by Mao, Cao, and Tan [21,22,23]. These authors evaluated dimethyl-, bathophenanthroline (**3a/c**, **4a/c**), and bathocuproine (**5a/b**) theranostic complexes of rhenium with monodentate pyridine ligands in order to simultaneously track and alter cancer cell metabolism [21,22] and epigenome [23]. Based on the original design of Coogan [45], the authors introduced bathophenanthroline species (**3**) containing a thiol-reactive chloromethylpyridyl moiety [21] or dichloroacetate derivatives of bathocuproine (**4**) [22] for mitochondria accumulation, displaying high cytotoxicity (IC_50_ value 0.5–4 μM) and selectivity against cancer cells. The complexes induce a cascade of mitochondria-dependent events (including mitochondrial damage, respiration inhibition, cellular ATP depletion, and caspase-dependent apoptosis), and due to their phosphorescence, the compounds could be utilized for real-time tracking of the morphological changes of mitochondria [21]. Similarly, rhenium phenanthroline complexes incorporating the clinical iron-chelating agent deferasirox (**5**) target the mitochondria as well and are able to alter key metabolic epigenetic species by relocating iron to the mitochondria. The complexes are highly potent against MDA-MB-231 cells with IC_50_ values of 0.4–0.8 μM [23]. 

The groups of Ye and Li [24] and Yu and Lo [25] have also studied the cytotoxicity of derivatized phen luminescent complexes of rhenium with pending substituents. The former authors described compounds incorporating the antimalarial drug artesunate (**6b**) and showed that the complexes can specifically accumulate in the mitochondria of human cervical carcinoma (HeLa) cells. Mechanism studies show that the bathophenanthroline derivative exhibits high cytotoxicity against different cancer cell lines (IC_50_ value 0.8–1.5 μM) and can induce both apoptosis and ferroptosis in HeLa cells through mitochondrial damage, caspase cascade, glutathione (GSH) depletion, glutathione peroxidase 4 (GPX4) inactivation, and lipid peroxidation accumulation [24]. Yu and Lo prepared similar complexes appended with perylene diimide or benzoperylene monoimide (**7a/c**) with excellent (photo)cytotoxic activity (IC_50_ value 0.4–0.7 μM) [25], but the vitamin B12 phen conjugates of Santoro are less effective [46]. 

The group of Wilson has studied the in vitro anticancer activity and in vivo biodistribution of *fac*-[Re(CO)_3_]^+^ aqua complexes, identifying the 2,9-dimethyl-1,10-phenanthroline derivative (**8c**, IC_50_ value 0.8–4 μM) as the most active one of their phen series of compounds [26]. The complex induces cytoplasmic vacuolization and cell death via a non-canonical pathway (apoptosis, necrosis, paraptosis, or autophagy), suggesting that a novel mode of action may be operative for this class of rhenium compounds. The in vivo biodistribution and metabolism analysis of the complex showed considerable metabolic stability, rendering it potentially suitable for in vivo applications [47]. The tricarbonyl rhenium isonitrile polypyridyl (TRIP, **9**) of the same laboratory also features the 2,9-dimethyl-1,10-phenanthroline diimine ligand [27,48]. This compound exhibits potent in vitro anticancer activity in a wide variety of cell lines (IC_50_ value 1.4–1.9 μM) and acts by triggering the accumulation of misfolded proteins, which causes endoplasmic reticulum (ER) stress, unfolded protein response, and apoptotic cell death in addition to mitochondrial fission. Like **8c**, x-ray fluorescence microscopy (XFM) has shown that **9** remains intact in vitro [49]. 

Within the studies above, Wilson also studied derivatized bipy *fac*-[Re(CO)_3_]^+^ complexes (**8a/b**), which were also found to be equally active to the phen compounds [26]. Our group has recently published a study describing a series of rhenium tricarbonyl bipy-based complexes that are non-toxic and are effective against colorectal carcinoma. One compound in particular, bearing 6-(bromomethyl)-2,2’-bipyridine (IC_50_ value 5 μM), was discovered to possess remarkable anticancer, anti-angiogenic, and antimetastatic activity in vivo, being effective at very low doses (1–3 μM) [50]. In vivo, at doses as high as 250 μM, the complex does not cause cardio-, hepato-, or myelotoxicity, and it exceeds the anti-tumor and anti-angiogenic activity of clinical drugs cisplatin and sunitinib malate, displaying a large therapeutic window [50]. When the compound is loaded on photoactivatable surface-functionalized marine diatom microalgae, its IC_50_ value is further improved [51,52,53]. Akin to the in vitro activity of these bipy complexes, several other species have been studied in the last seven years [30,31,32,33,34]. Of particular relevance are the derivatives of Alberto (**10a/b**), capable of diverting doxorubicin from the nucleus to the mitochondria and displaying excellent toxicity toward HeLa cells (IC_50_ value 0.34 μM) [28]—the *N*-heterocylic carbene complexes (**11a/b**) of Falasca, Massi, and Simpson [29,54] and the diseleno-ether compound (**12**) of Collery [55,56,57,58,59]—both studied extensively in vivo.

### 2.2. Homonuclear Complexes

Homonuclear multimetallic *fac*-[Re(CO)_3_]^+^ complexes have recently attracted substantial interest. The arrangement of two or more *fac*-[Re(CO)_3_]^+^ fragments into one well-defined molecular structure has the potential to improve the well-established biological properties of the core, such as improved cellular uptake, by increased lipophilicity of the molecules and, in turn, to improve the cytotoxicity properties of the same [10,60,61]. The majority of the homonuclear complexes reported to date are those linked through axial pyridine ligands. The groups of Sakthivel and Manimaran have been particularly active in this field. The approach the authors took is that of the self-assembly of precursor rhenium carbonyl complexes with different functionalized monodentate organic spacers as ditopic pyridyl ligands to M_2_L_2_ type dinuclear species (where M = *fac*-[Re(CO)_3_]^+^) [62,63,64,65,66]. These species form either metallacycles [62,63,64] or metallastirrups [65,66], the fundamental differences being that, in the former, the rhenium centers are connected by two flexible ditopic ligands, retaining one free coordination site, and in the latter, all three coordination sites are shared by the metals via a single flexible ligand and either two μ-bridges or one rigid bidentate unit. Most active complexes selectively inhibit certain cancer cells with IC_50_ values comparable to cisplatin [62,63,66], and in specific cases, the anticancer activity of the compounds is attributed to the induction of early apoptosis [62]. 

**Figure 2:**
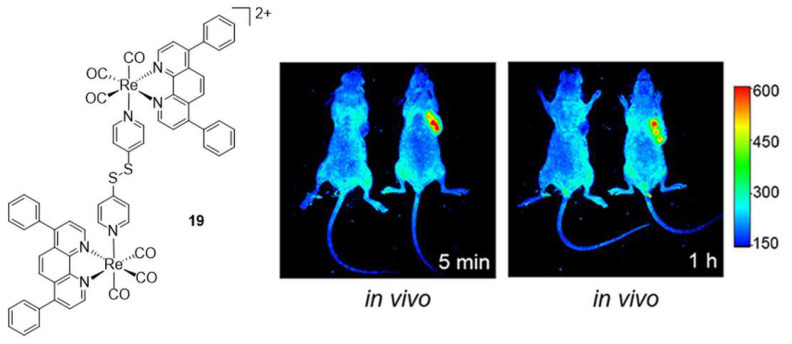
Structure of **19** and in vivo fluorescence emission of the compound after intratumoral injection in a mouse xenograft model (1 mg/kg, right mouse in both panels). The molecule was dissolved in 6% polyethylene glycol 400, 3% ethanol, and 1% Tween 80 in phosphate-buffered saline (PBS). The excitation wavelength is 430 nm, and the maximum emission wavelength is 600 nm. Color scale refers to emission intensity (a.u.). Figure readapted with permission from publisher [36].

**Figure 3:**
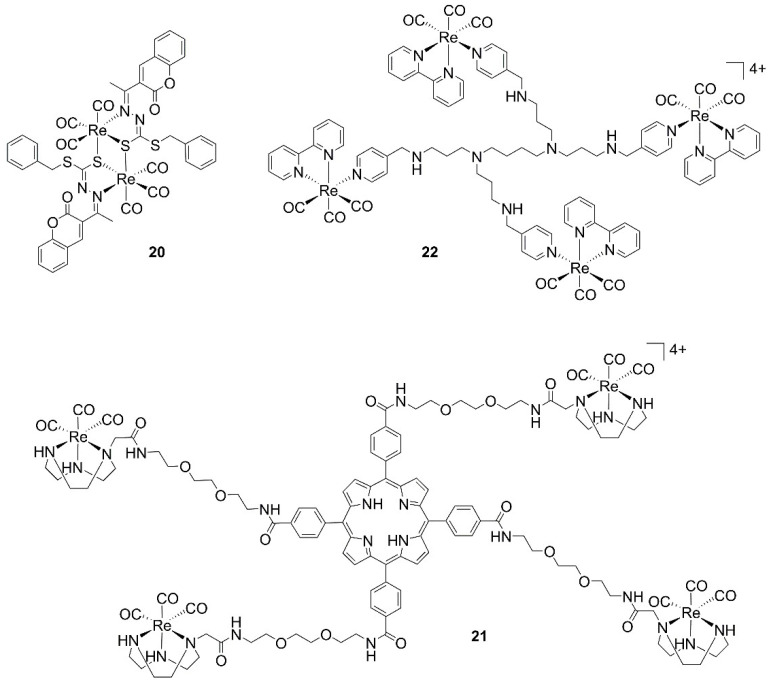
Selected structures of homonuclear anticancer complexes.

Tan and Mao have reported a series of diimine dinuclear complexes bridged via ditopic 4,4′-derivatized pyridines, namely 1,2-di(pyridin-4-yl)ethane [67], 4,4′-azopyridine, or 4,4′-dithiodipyridine (**19**, Figure 2) [36]. The authors found that the 1,2-di(pyridin-4-yl)ethane dinuclear complexes are more potent than the mononuclear counterparts (IC_50_ ca. 2 μM and 2–12 μM, respectively), confirming a synergistic effect of the two metal ions, and that increased lipophilicity of the bidentate diimine ligands enhances cellular uptake, leading to improved anticancer efficacy. Complexes of lower lipophilicity localize in lysosomes and induce caspase-independent apoptosis, whereas higher lipophilicity compounds target mitochondria and induce caspase-independent paraptosis in cancer cells [67]. Similarly, 4,4′-azopyridine and 4,4′-dithiodipyridine bridged homonuclear species accumulate in mitochondria, where they can react with glutathione (GSH), cause oxidative stress, and disturb GSH metabolism. Interestingly, these latter species simultaneously induce necroptosis and caspase-dependent apoptosis and inhibit tumor growth in nude mice bearing carcinoma xenografts, showing very low in vitro IC_50_ values (ca. 1 μM against different cell lines) [36]. In vivo, the mouse xenograft model, **19**, is effectively retained in tumors as revealed by fluorescence emission spectroscopy (Figure 2). Liang He’s group has used the same molecular design of Tan and Mao, varying the diimine bidentate ligand with a *9H*-pyrido [3,4*-b*]indole unit [68]. The dinuclear complexes show potent anticancer activities toward several cancer cells (IC_50_ values 2–5 μM) and marked inhibitory effects (16-fold higher activity than cisplatin) against cisplatin-resistant human lung carcinoma cells (A549R) in addition to substantial phototoxicity (425 nm irradiation). Mechanistic studies have shown that complexes can induce overproduction of reactive oxygen species (ROS), which leads to lysosomal membrane permeabilization and subsequent cell apoptosis. The groups of Crouse and Policar have instead prepared a dinuclear 1:2 complex of the Schiff base, *N’*-[1-(2-oxo-2H-chromen-3-yl)-ethylidene]-hydrazinecarbodithioic acid benzyl ester with *fac*-[Re(CO)_3_]^+^ fragments. The ligand induces a centrosymmetric dimeric complex with the metal cores linked by Re–S–Re bridges (**20**, Figure 3). Cytotoxicity assays revealed that the compound is active against human breast adenocarcinoma cancer cell lines MDA-MB-231 and MCF-7 [37].

Higher-order homonuclear multimetallic *fac*-[Re(CO)_3_]^+^ complexes have been prepared by Gianferrara, Gasser, and Vilar [38] and the Smith group [39]. The former authors described phototoxic neutral fourfold-symmetric porphyrins derivatized with four [1,4,7]-triazacyclononane rhenium complexes connected to the porphyrin macrocycle through hydrophilic spacers containing the ethylenedioxy groups (**21**, Figure 3). Although the compound is only moderately toxic in the dark against HeLa (cervical cancer), H460M2 (non-small-cell lung carcinoma), and HBL-100 (non-tumorigenic epithelial cells), its in vitro anticancer effects are greatly improved when irradiated (up to 71-fold, with IC_50_ values as low as 0.5 μM). The activity of the complex is associated with ^1^O_2_ production and selective interaction with G-quadruplex DNA. On the other hand, Smith reported tri- and tetranuclear dendritic complexes in a study aimed at exploring the multivalency offered by dendrimers as a way to improve site-specific drug delivery and reduced systemic exposure to the drug [39]. The compounds display only moderate biological activity against different cancer cell lines, with the tetranuclear system (**22**, Figure 3) showing selective cytotoxicity towards the tumors. The authors performed in-depth mechanistic studies and found that the species influence programmed cell death in vitro. They are able to inhibit the soluble form of the programmed cell death Fas receptor in malignant cells, allowing the Fas domain to receive the apoptotic signal through an extrinsic pathway. Moreover, the tetranuclear complex modulates and augments intracellular levels of the pro-apoptotic Bax-α product, leading to cell death [39]. Interestingly, the polynuclear complexes **21** and **22** are more active than the corresponding mononuclear compounds, further substantiating the potential synergistic effect of the cores to improve the biological properties and cytotoxicity properties of homonuclear multimetallic *fac*-[Re(CO)_3_]^+^ complexes.

### 2.3. Heteronuclear Complexes

An important strategy being recently employed in the development of novel anticancer agents is the incorporation of two distinct metal centers to form heterobimetallic systems. In this way, the biological and chemical features of two metal centers may potentially be combined into one well-defined molecular structure. In heterobimetallic systems, the presence of two different metals provides an opportunity to utilize their desirable and distinct features simultaneously. These systems include combinations of rhenium carbonyl species with different metals [46,69,70,71,72], but in particular Fe [40,73,74], Pt [41,42,43,44,75], and Au [76,77,78,79]. In such systems, which may be less susceptible to drug resistance mechanisms, a synergistic effect is often observed, resulting in enhanced cytotoxicity as compared to mononuclear analogues. 

**Figure 4:**
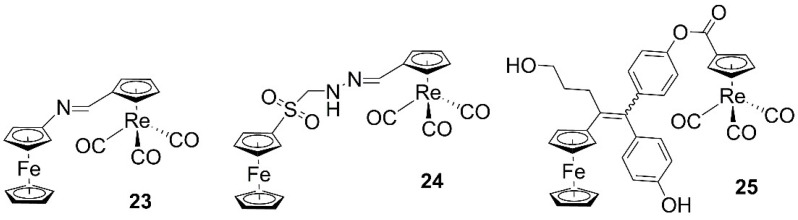
Structures of heteronuclear iron-rhenium anticancer complexes.

Iron-rhenium multifunctional compounds evaluated in anticancer studies are predominantly those of ferrocenyl derivatives. Isomeric hybrid ferrocenyl/cyrhetrenyl aldimines of Klahn, Arancibia, and López (**23**, Figure 4) show cytotoxic activities against human adenocarcinoma breast (MCF7 and MDA-MB-231) and colon (HCT-116)] cell lines, with greater inhibitory growth effect than cisplatin in the triple negative MDA-MB 231 and in the cisplatin resistant HCT-116 cell lines [40]. The authors later confirmed the synergistic effect of these type of complexes with ferrocenyl sulfonyl hydrazide derivatives (**24**, Figure 4) [73]. In vitro cytotoxicity studies of the compounds against the same cell lines revealed that the ferrocenyl complex has no significant activity (IC_50_ > 100 μM), while the rhenium heterobimetallic systems is active in all three lines. The growth inhibition potency of the hybrid compound is similar than that of cisplatin, but it is less toxic than cisplatin in normal and non-tumoral BJ fibroblasts. Similarly, ferrocifen complexes tagged with cyrhetrenyl (**25**, Figure 4) preserve high cytotoxicity against MDA-MB-231 cells with IC_50_ values in the range 0.32–2.5 μM and mainly localize in the cell nucleus [74].

**Figure 5:**
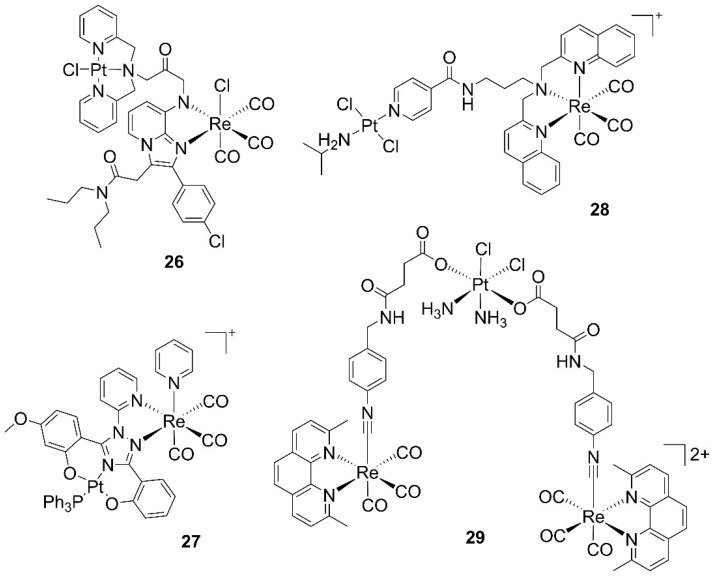
Structures of heteronuclear platinum–rhenium anticancer complexes.

Heterobimetallic platinum-rhenium species recently reported are of two types: either incorporating Pt(II) coordination complexes [41,42,43] or redox-active Pt(IV) prodrugs [44,75]. In 2014, Margiotta and Natile reported the synthesis, characterization, and in vitro evaluation of the first mixed platinum(II) and rhenium(I) compound of the bifunctional chelating, translocator protein (TSPO) targeting, and CB256 ligand (**26**, Figure 5) [41]. The heterobimetallic metal complex shows cellular uptake and is able to induce apoptosis in C6 glioma cells. The mechanism of the antiproliferative activity is associated with collapse of the mitochondrial membrane potential and interference with the cell-cycle progression of glioma C6 cells. Coordination of the metals to CB256, however, diminishes the cytotoxicity of the free ligand towards C6, A2780, and A2780cisR tumor cell lines and reduces the affinity of the same towards TSPO. Bertrand introduced mixed metal complexes of platinum and rhenium bound to a modified bis[2-(hydroxyphenyl)-1,2,4-triazole] ligand (**27**, Figure 5) and evaluated the anticancer properties of the compounds on a panel of human cancer cells (MDA-MB-231, MCF-7, and A2780), as well as on a nontumorigenic cell line (MCF-10A) [42]. The molecules show low micromolar activities (IC_50_ values of 1–2 μM) and represent rare examples of highly active, but hydrolytically stable, Pt(II) complexes.

Gasser, Quiroga, and Paulo developed an anticancer agent with chemotherapeutic and photosensitizing properties by connecting a non-conventional *trans*-chlorido Pt(II) complex to a photoactive Re tricarbonyl unit (**28**, Figure 5) [43]. The complex shows dark- and enhanced photo-toxicity with IC_50_ values in the low μM range, with similar activity against the A2780 and A2780R cell lines, indicating that the heterobimetallic complex can significantly circumvent cisplatin cross-resistance. This multimodal theranostic approach was also realized with redox-active Pt(IV) prodrugs. Paulo and Ravera modified chitosan with Pt(IV) and Re(I) tricarbonyl complexes but found low to moderate cytotoxic activity of the conjugate, in line with the values of the constituent complexes [75]. Similar results were obtained by Wilson with a tricarbonyl rhenium isonitrile polypyridyl complex coupled with cis- and oxaliplatin Pt(IV) prodrugs (**29**, Figure 5) [44]. In the dark, the molecules exhibit modest anticancer activity against wild-type (A2780) and cisplatin-resistant (A2780CP70) ovarian cancer cell lines. Upon irradiation with 365 nm light, a 2–3-fold enhancement in cytotoxicity against A2780 cells for both conjugates is observed. Within the cisplatin-resistant A2780CP70 cell line, however, the photoactivated drug candidates show mixed results.

**Figure 6:**
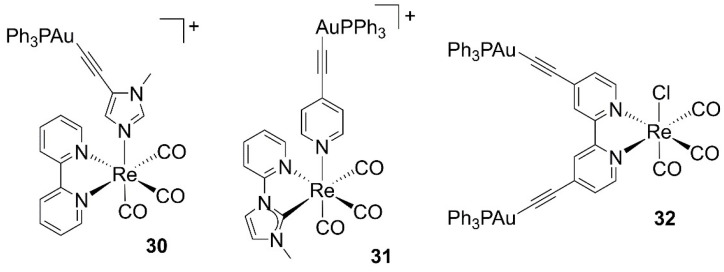
Structures of heteronuclear gold–rhenium anticancer complexes.

Work on bioactive heterobimetallic gold–rhenium conjugates comes from the laboratory of Fernandez-Moreira and Gimeno [76,77,78,79]. In 2014, the authors reported the first series of heterometallic *fac*-[Re(bipy)(CO)_3_(L-AuPPh_3_)]^+^ complexes (where L is a monodentate aromatic ligand, **30** in Figure 6) in a study aiming at the preparation of antiproliferative trackable drugs [76]. Cytotoxicity studies against human A549 lung cancer cells identified the alkynyl–phosphine–gold fragment as the moiety essential for the bioactivity of the molecules. These heterometallic Re(I)/Au(I) derivatives show IC_50_ values one order of magnitude lower than their analogous monomeric Re(I) complexes, again substantiating the synergistic effect in mixed metal species. The effect may be possibly due to the different bio-distribution behavior of the monometallic and heterometallic families. Whereas the monometallic Re(I) species show some general cytoplasmatic staining and mitochondrial accumulation, the heterometallic Re(I)/Au(I) derivatives accumulate in the nucleus. Substitution of the alkynyl for a ditopic P,N-donor linker drastically reduces the cytotoxicity of the molecules, with hetero-trimetallic species almost twice as toxic as the heterobimetallic analogues [77]. Variation of the bidentate chelate of the *fac*-[Re(CO)_3_]^+^ core affects less the bioactivity of the molecules. Thus, substitution of bipy for imidazole pyridine-based carbenes (**31**) preserves the antiproliferative activity, with only the heterobimetallic species being effective against tumor lung A549 cells. A necrotic process seems to be the preferred cell death mechanism of these perinuclear-accumulating derivatives, whose IC_50_ values can be improved nearly five times if irradiated at 405 nm [78]. A final series of neutral and cationic heterotrimetallic complexes of the type *fac*-[Re(CO)_3_(bipy(CC)_2_-(AuPPh_3_)_2_)X]^n^ (where bipy(CC)_2_ is 4,4′-alkynyl-2,2′-bipyridine, **32**), shows a generalized selectivity toward HeLa (cervix cancer) cells over A549. Once again, the effect may be possibly due to the different bio-distribution behavior of the molecules. Whereas the complexes are randomly distributed in A549 cells, they locate close to the cellular membrane in HeLa cells, possibly explaining their cellular selectivity when it comes to the antiproliferative activity displayed in the different cell lines [79].

## 3. Antibiotic Complexes

**Figure 7:**
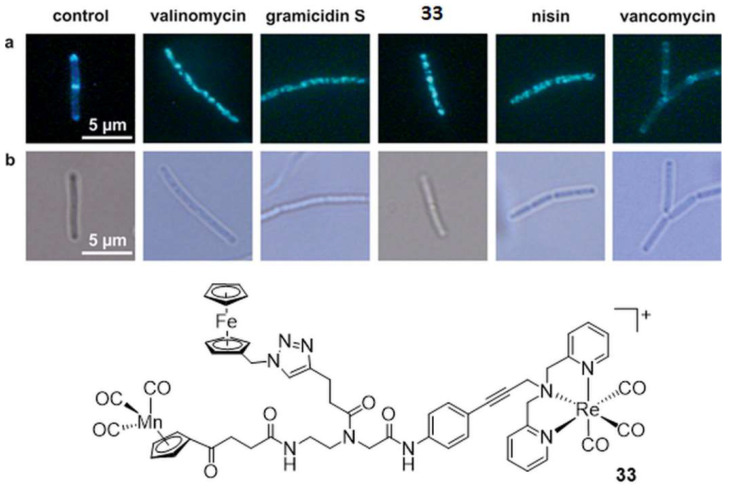
Top: influence of hetero-tri-organometallic compound **33** of Metzler-Nolte and Bandow on the *B. subtilis* membrane and cell wall integrity. (**a**) GFP-MinD (green fluorescent cell division protein) localization after treatment with molecule **33** and approved antibiotics (valinomycin, gramicidin S, nisin, and vancomycin). Like valinomycin, gramicidin S, or nisin (positive control), **33** leads to GFP-MinD delocalization, demonstrating membrane depolarization by the molecule. Note that vancomycin here represents the negative control. (**b**) Light microscopy images corresponding to panel a. Bottom: structure of the hetero-tri-organometallic molecule **33**. Figure readapted with permission from publisher [80].

The evaluation of the antibiotic potentials of rhenium carbonyl complexes began in 2013 with the seminal study of Metzler-Nolte and Bandow [80]. The groups identified two hetero-tri-organometallic compounds, with potent activity (MIC 1.4 µM) against Gram-positive bacteria including multi-resistant *Staphylococcus aureus* (MRSA), consisting of a peptide nucleic acid backbone with an alkyne side chain, substituted with a cymantrene, a [(dipicolyl)Re(CO)_3_] moiety, and either a ferrocene or a ruthenocene unit. Comparative proteomic analysis revealed the bacterial membrane as an antibiotic target structure of the molecules (**33**, Figure 7). Both hetero-tri-organometallic compounds interfere and disrupt several essential cellular processes such as respiration and cell wall biosynthesis taking place at the membrane (Figure 7). Crucially, a systematic structure–activity relationship study against various pathogenic bacteria, including MRSA, proved that the [(dipicolyl)Re(CO)_3_] moiety is the essential part for the antibacterial activity of the trimetallic complexes [81]. The other metallic units can be replaced without substantially affecting the antibiotic properties of the constructs.

The report of Metzler-Nolte and Bandow arguably sparked what is becoming a thriving field of rhenium carbonyl research, with several related papers appearing since the publication of their study. As is the case for anticancer species, the overwhelming majority of rhenium carbonyl complexes tested to date are those of the *fac*-[Re(CO)_3_]^+^ core. We are aware of only two reports dealing with a different carbonyl unit, namely *cis*-[Re(CO)_2_]^+^ [82,83] but these are discussed later. Most tricarbonyl complexes unfortunately do not show antibiotic properties at low concentrations (defined here as MIC < 10 µg/mL) [84,85,86,87,88,89,90,91,92,93], but the ones that do are remarkable. In 2017, Metzler-Nolte and Bandow reported a family of *fac*-[Re(CO)_3_]^+^ N-heterocyclic carbene complexes containing unsubstituted benzimidazol-2-ylidene and phenanthroline, with MIC values against Gram-positive bacteria between 0.7–2 µg/mL [94]. The mechanism of action of these molecules is yet undetermined. In 2019, Frei and Blaskovich reported the antibacterial activity of rhenium bisquinoline complexes displaying light-induced antibiotic properties against drug-resistant *S. aureus* and *E. coli* strains with MIC values between 0.25–8 µg/mL when photo-irradiated [95]. The molecules appear to act via two distinct mechanisms, one of which is associated with ROS production possibly leading to destabilization of Fe–S clusters and increased aminoglycoside uptake.

In 2020, Sovari reported a series of *fac*-[Re(CO)_3_]^+^ species bearing arylcoumarin ligands and tested all compounds for their antimicrobial efficacy against both bacteria and fungi [96]. Whereas the arylcoumarin ligands are virtually inactive against the human-associated pathogens, most of the corresponding complexes show remarkable antibacterial potency (MIC values of 0.8 µg/mL). Several compounds exhibit in vivo activity in nanomolar concentrations against MRSA and *Enterococcus faecium*, two organisms commonly associated in hospital infections and intrinsically resistant to several antibiotics [97]. The same in vivo studies (zebrafish-MRSA infection model) showed that the complexes with anti-staphylococcal/MRSA activity are non-toxic and capable of increasing infected fish survival rate up to 100% while markedly reducing bacterial burden. The mechanism of action of the molecules is unknown, but it is known that they do not affect bacterial cell membrane potential. 

**Figure 8:**
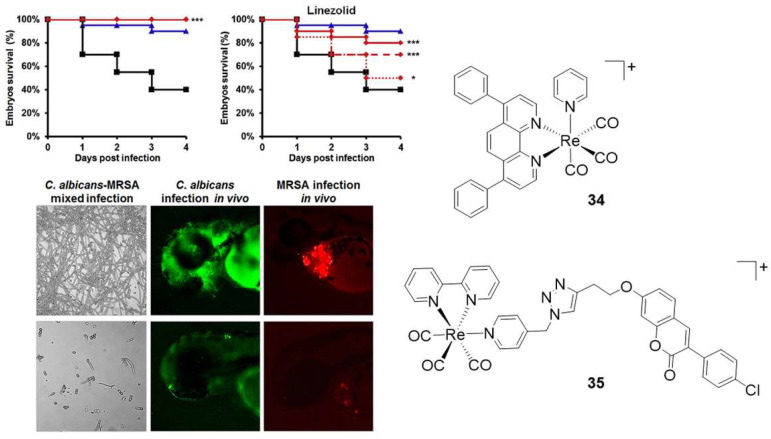
Top left: Kaplan–Meier survival curves of the MRSA- or *C. albicans*-infected zebrafish embryos treated with molecules **34** or **35** (top-left graph; molecules show identical fish survival response) in comparison with that of the approved drug Linezolid (top-right graph). In the Kaplan–Meier survival curves, the red line = ½ × MIC; blue line = non-infected control; black line = infected control; dashed red line = 1 × MIC and dotted red line = 2 × MIC. Note that in the top-left Kaplan–Meier survival curve, all red lines overlap, indicating that **34** and **35** are safe and non-toxic at all tested concentrations (i.e., all MIC values). Bottom left: in vitro and in vivo antimicrobial effects of molecule **34** in *C. albicans*–MRSA mixed infections; lethal *C. albicans* infection and lethal MRSA infection. The green and red fluorescence intensity of labelled microbes (as a measure of infection burden) is markedly reduced upon treatment with **34**. Asterisks stand for statistical significance in the survival rates between treated and untreated embryos (* *p* < 0.05; *** *p* > 0.001). Figure readapted with permission from publisher [98].

The same researchers published this year a more comprehensive study aiming also at understanding, via a structure–activity relationship (SAR) analysis, what molecular and structural parameters are responsible for the antibiotic activity (or lack thereof) of *fac*-[Re(CO)_3_]^+^ complexes [98]. Their study included 57 diimine species with varying ligands, charge, and lipophilicity and allowed the identification of potent and non-toxic complexes active in vivo against *S. aureus* infections at MIC doses as low as 300 ng/mL (**34** and **35** in Figure 8), as well as against *C. albicans*-MRSA mixed co-infection. The compounds are capable of suppressing the *C. albicans* morphogenetic yeast-to-hyphal transition, eradicating fungal–*S. aureus* co-infection, while showing no sign of cardio-, hepato-, or hematotoxicity or teratogenicity. Overall, the molecular analysis (which also included all other published complexes) indicates that all active and non-toxic complexes are those bearing highly lipophilic diimine ligands and an overall positive charge. These features appear common to both effective antibacterial and antifungal compounds. The authors hypothesized that these features are important for their interaction with phosphatidylglycerol and cardiolipin anionic lipids [98].

## 4. Rhenium Dicarbonyl Complexes: Is There a Future for These Species?

**Figure 9:**
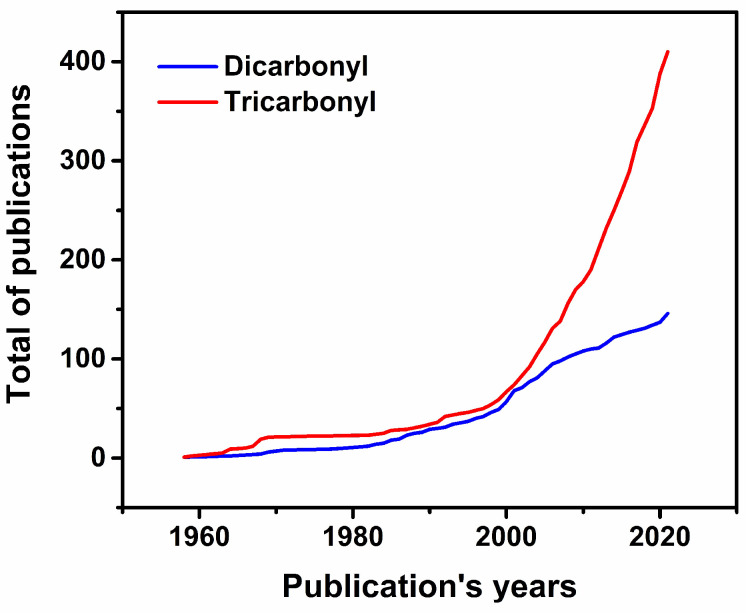
Progressive sum of publications of rhenium dicarbonyl (blue) and tricarbonyl (red) complexes as a function of the year. Queries were performed on 23 November 2021 with Scifinder employing the following key words: “rhenium dicarbonyl complexes” and “rhenium tricarbonyl complexes”.

It is evident from what was discussed above that tricarbonyl complexes of rhenium dominate the medicinal chemistry of the metal ion. We believe that there are two main reasons contributing to this. First, the relative ease of preparation of molecules, which can be obtained in one or two synthetic steps, often from commercially available starting materials. The compounds are very stable both in the solid state and in solution, and they have favorable spectroscopic characteristics that aid with their analysis. Complexes of the *fac*-[Re(CO)_3_]^+^ core are diamagnetic, with energetically isolated CO stretching vibrations whose frequency can be mathematically predicted [99,100]. Second, the unique photophysical and luminescent properties of the complexes allow the combination of diagnostic and therapeutic purposes, thereby permitting the molecules to be tracked within cells [7,101]. In comparison, complexes of the *cis*-[Re(CO)_2_]^+^ core are rarer and consequently less studied in biological and medicinal applications. One of the underlying reasons is the relative difficulty of preparing molecules of this core, whose chemistry is dominated by π-acid ligands, necessary to stabilize it. In recent years, however, several groups have begun investigating in detail the chemistry of the dicarbonyl core. Publications of this core are slowly gaining momentum (Figure 9), and a few examples of anticancer and antibiotic complexes are now available. Before discussing these examples, we thought it would be useful to briefly review the synthetic procedure currently known for the preparation of rhenium dicarbonyl complexes. We focus on studies achieved during this last decade, and we intend the following section as a quick “reference book” for readers interested in this chemistry. Our analysis has evidenced the existence of the five main synthetic decarbonylation reactions.

## 5. Preparation *cis*-[Re(CO)_2_]^+^ Complexes via Decarbonylation Reactions

### 5.1. Trimethylamine N-Oxide Decarbonylation

Trimethylamine N-oxide (Me_3_NO) reacts selectively and irreversibly with a rhenium-metal-bound CO liberating CO_2_, NMe_3_, and a coordination site on the metal ion then occupied by a coordinating ligand or solvent molecule. The general reaction below follows a second-order rate law consistent with a bimolecular mechanism [102]. The procedure

*fac-*[Re(CO)_3_**L**_3_]^n^ + ONMe_3_ + *L* ⟶ *cis-*[Re(CO)_2_**L**_3_*L*]^n^ + CO_2_ + NMe_3_

has been successfully applied to both rhenium tricarbonyl and pentacarbonyl complexes. When *fac*-[Re(CO)_3_]^+^ complexes are used, best results are achieved starting with a diimine phosphine or phosphite compound in either acetonitrile (MeCN) or in the presence of an halide X (e.g., Cl^-^), which act as the entering “*L*” by replacing CO. Examples of this synthetic approach have been recently described by Ko [103,104], Ishitani [105,106], Felton [107], Visser [108], and Miller and Dempsey [109] for the preparation of *cis,trans*-[Re(CO)_2_(diimine)(PR_3_)(*L*)]^+^ complexes where (*L* = MeCN or Cl^-^), starting from the corresponding *fac*-[Re(CO)_3_(diimine)(PR_3_)]^+^ complexes. The only example we are aware of in which a [Re(CO)_5_]^+^ precursor was decarbonylated with Me_3_NO was described by Mondal [110] in the reactions of rhenium pentacarbonyl halides and the thioarylazoimidazole SNN pincer ligands. The complexation reaction yields directly *cis,mer*-[Re(CO)_2_(SNN)X] complexes.

### 5.2. Photo-Decarbonylation

As the name of the reaction implies, UV-light is used to generate an excited state in *fac-*[Re(CO)_3_**L**_3_]^n^ complexes, which promotes the release of one equivalent of CO, subsequently replaced by *L*. The details and mechanisms of the general reaction below are well-understood and, since they were recently reviewed [101], are not discussed here.

*fac-*[Re(CO)_3_**L**_3_]^n^ + hν + *L* ⟶ *cis-*[Re(CO)_2_**L**_3_*L*]^n^ + CO

*fac*-[Re(CO)_3_(diimine)(L)]^+^ complexes (where L = PR_3_, MeCN, NHC, CN^-^ or halide) are often studied in this context [111,112,113,114,115,116], but obviously critical for the reaction is the presence of a coordinating *L*. Ishitani, Onda, Miyasaka, and coworkers, e.g., have shown that irradiation of *fac*-[Re(CO)_3_(bipy)Cl] in THF leads to the rearrangement of the CO ligands to corresponding *mer*-isomer, but the same reaction in MeCN leads to the *cis,trans*-[Re(CO)_2_(bipy)Cl(MeCN)] species [111]. A few cyclopentadiene (η-C_5_H_5_) rhenium tricarbonyl complexes undergoing photo-decarbonylation have also been reported [117,118,119]. As an example, Hill studied the photo-decarbonylation of cyrhetrenyl in THF in the presence of carbon disulfide and triphenylphosphine. The reaction of the photo-excited intermediate with PPh_3_ and CS_2_ leads to three different rhenium dicarbonyl complexes: [Re(CS)(CO)_2_(η-C_5_H_5_)], [Re(PPh_3_)(CO)_2_(η-C_5_H_5_)] and the bimetallic carbido [(µ-C){Re(CO)_2_(η-C_5_H_5_)}_2_] complex [118].

### 5.3. Redox-Mediated Decarbonylation

In this reaction (see below) the oxidation state of a *fac*-[Re(CO)_3_]^+^ complex is increased in order to diminish the metal to CO π-back bonding and consequently weaken the metal–CO bond. The presence of halides is fundamental in the reaction in order to stabilize the higher oxidation state of the rhenium ion. To the best of our knowledge, the only redox-mediated decarbonylation of rhenium tricarbonyl is the procedure of Abram [120], who described the oxidation of *fac*-[Re(I)(CO)_3_Br_3_]^2-^ with bromine to the *cis*-[Re(III)(CO)_2_Br_4_]^-^ anion. This complex then served as the synthon for several reactions described during the last decade.

*fac-*[Re(CO)_3_**L**_3_]^n^ + oxidizing agent + *L* ⟶ *cis-*[Re(CO)_2_**L**_3_*L*]^n+1 or 2^ + CO

The group of Alberto, in particular, has described the redox reactivity and ligand substitution chemistry of the one electron reduced congener (Et_4_N)_2_[Re^II^(CO)_2_Br_4_] as a stable 17-eletron species [121,122,123]. Our group has studied the carbon-monoxide-releasing properties of these compounds [124,125] and conjugated them to cyanocobalamin, making them pharmaceutically acceptable [126,127]. These molecules display efficient therapeutic protection toward ischemia-reperfusion injury and anti-platelet activity [128]. More recently, we introduced new synthetic routes to aerobically stable and substitutionally labile α-diimine rhenium(I) dicarbonyl complexes lacking π-acid ligands [129].

### 5.4. Thermal Decarbonylation

This type of decarbonylation reaction is mainly performed in the solid state, starting from *fac*-[Re(CO)_3_]^+^ complexes, and it involves activation of the core with high temperature (e.g., 275–300 °C), under either vacuum or a constant flow of an inert gas. However, examples of the same reaction performed in solutions are known [130,131]. Common products of the solid state thermal decarbonylation are *cis*-[Re(CO)_2_(NN)_2_]^+^ (where NN are bipy or phen derivatives) [132], *cis,mer-*[Re(CO)_2_(terpy-κ^3^N)L]^n^ [133,134,135,136,137], or similar pincer complexes [138,139,140]. The general reactions below depict these transformations.

*fac-*[Re(CO)_3_**NN**L]^n^ + *NN* + heat ⟶ *cis-*[Re(CO)_2_**NN**(*NN*)]^n^ + CO + L

*fac-*[Re(CO)_3_(terpy-κ^2^N)L]^n^ + heat ⟶ *cis,mer-*[Re(CO)_2_(terpy-κ^3^N)L]^n^ + CO

Interestingly, [Re(κ^3^N-Rterpy)(CO)_2_(Br)] complexes are panchromatic, whereas bidentate tricarbonyl derivatives are scarcely absorbed in the visible region [137]. Another example of solid-state products of note are the [4 × 1] square and a [3 × 1] triangular κ^3^N-terpyridine rhenium dicarbonyl assembly achieved by head-to-tail bonding reported by Zaccheroni and Hanan [136]. In solution, the thermal reaction is best suited if CO is replaced by a phosphine (PR_3_) ligand. However, a strong π-acid already coordinated to the *fac*-[Re(CO)_3_]^+^ core is needed in order to activate the carbonyl complex towards the substitution reaction [130,131]. 

### 5.5. Nitrosylation

The general reaction depicted below entails the direct substitution of a CO ligand with a nitrosonium ion yielding complexes of the *fac-*[Re(CO)_2_(NO)]^2+^ core. This transformation is well-known for cyclopentadienyl rhenium complexes [141,142] or rhenacarboranes [143,144], with nitrosonium tetrafluoroborate, NOBF_4_, being the preferred reagent. Particularly interesting are the rhenacarborane complexes developed by Jelliss and

*fac-*[Re(CO)_3_**L**_3_]^n^ + *N*O^+^ ⟶ *fac-*[Re(CO)_2_(*N*O)**L**_3_]^n+1^ + CO

pruitt as central nervous system drug-delivery agents [144]. The authors showed that nitrosylation of tricarbonyl rhenacarboranes precursors is possible for a variety pendant functional groups (chloride, bromide, iodide, benzyl ether and alcohol), except for azide where significant decomposition of the complex is observed [143]. The corresponding ^125^I derivatives permeate the blood–brain barrier with brain uptake exceeding the maximum uptake of other prototypes [144]. Finally, our group has recently reported the nitrosylation of rhenium tricarbonyl complexes bearing α-diimine ligands [83]. The reaction between *fac*-[Re(CO)_3_(NN)Br] precursors and an excess of NOBF_4_ in DCM leads to dicarbonyl-nitrosyl *fac*-[Re(CO)_2_(NO)(NN)Br]^+^ complexes in high yields. 

## 6. Anticancer and Antibiotic Dicarbonyl Complexes

As mentioned in the previous section, examples of rhenium dicarbonyl complexes evaluated for their anticancer and antibiotic properties are relatively rare compared to tricarbonyl species. However, encouraging data are available for these species. The first examples of a *cis*-[Re(CO)_2_]^n^ evaluated for its anticancer properties actually came from our group in a study aimed at exploring organometallic cobalamin anticancer derivatives for the targeted prodrug delivery via transcobalamin-mediated uptake [145]. Surprisingly, among the metal complexes evaluated (including cisplatin and other Pt, Ru compounds), the 17-electron *cis*-[Re(CO)_2_(HCCbipy)Br_2_]^2+^ species (where HCCbipy = 4-ethynyl-2,2’-bipyridine) was the most active species, showing an IC_50_ value against MCF-7 cells of 1.4 μM (IC_50_ value of cisplatin = 3.7 μM). Within the same context, Wilson studied fifteen water-soluble rhenium compounds of the general formula *fac*-[Re(CO)_3_(NN)(PR_3_)]^+^, and evaluated them as photoactivated anticancer agents in human cervical (HeLa), ovarian (A2780), and cisplatin-resistant ovarian (A2780CP70) cancer cell lines. Several compounds exhibit a cytotoxic response (IC_50_ value of ca. 5 μM) upon irradiation with minimal toxicity in the absence of light. The authors investigated the nature of the photoinduced cytotoxic product and found that the phototoxic response may result from the presence of *cis*-[Re(CO)_2_(NN)(PR_3_)(H_2_O)]^+^ species in conjunction with the released CO as well as the production of ^1^O_2_ [115]. Godoy and Metzler-Nolte reported a series of cyclopentadienyl dicarbonyl complexes carrying phophine ligands of general formula [(η_5_-C_5_H_4_CHO)Re(CO)_2_PR_3_] [119]. The authors tested their complexes towards colon and pancreatic cancer cell lines and found that only the most lipophilic compound showed moderate biological activity. 

More recently, Visser reported the aquation of rhenium(I) dicarbonyl complexes of the general formula [Re(CO)_2_(NN)(PR_3_)(Cl)], where NN are phenanthroline derivatives and PR_3_ water-soluble phosphorous ligands in relation to their cell toxicity and bioavailability [108]. The authors found that at Cl^-^ concentration in biological environments (blood plasma, cell cytoplasm, and cell nucleus), the major species are the corresponding rhenium aqua complexes. Although several of these dicarbonyl complexes were not highly cytotoxic, the complete library of these compounds has not yet been subjected to thorough biological study. Antibiotic dicarbonyl complexes are ever rarer. As mentioned in Section 3, we are aware of only two reports dealing with *cis*-[Re(CO)_2_]^+^ species [82,83]. Kottelat prepared different isocyanide (CNR) *cis*-*mer*-[Re(CO)_2_(CNR)_3_Br] species and tested them against *E. coli* [82]. These rhenium complexes are not active, showing an MIC value > 1 mg/mL. Similarly inactive are the *fac*-[Re(CO)_2_(NO)(NN)Br]^+^, which we recently reported [83]. The antimicrobial activity of several different complexes, determined against four Gram-negative bacteria (*E. cloaceae*, *K. pneumoniae*, *A. baumanii*, and *P. aeruginosa*), two Gram-positive bacteria (*S. aureus* MRSA43300 and *S. aureus*) and two fungi (*C. albicans* and *C. auris*), indicates that none of the complexes has antibiotic properties, with MICs > 100 μM. 

## 7. Conclusions

In this brief review, we have summarized organometallic rhenium tri- and dicarbonyl compounds with antibiotic and anticancer activity published in the last seven years. We have selected in most cases only active complexes (defined as having in vitro IC_50_ and MIC values ≤ 5 μM or <10 µg/mL, respectively) and highlighted, where possible, their modes of action. The majority of anticancer rhenium complexes are believed to act by reacting with available biomolecules, thus forming traditional metal–ligand bonds, but a few exceptions exist. In addition to these, photoactive complexes have been increasingly reported. However, it must be said that in most cases, the underlying mechanisms of toxicity (both in terms of the anticancer and antibiotic properties of the compounds) are not known or not fully elucidated, but still most of the compounds discussed in this review show potent anticancer effects with IC_50_ values exceeding the activity of cisplatin. Similarly, antibiotic complexes are often more effective than approved drugs. Several compounds also show a synergistic action with other metal ions. We are not aware of clinical trials involving rhenium complexes at this time, and there are still no non-platinum metal-based drugs approved for cancer treatment. Undoubtedly, more biological investigations, both in vitro and in vivo, will allow the further advancement of this class of compounds. In particular, it would be useful if, along with the initial screenings based on the determination of the usual concentration inhibiting the growth of 50% of cancer cells in culture, the toxicity of the compounds would be regularly evaluated on normal healthy cells or tissues. Furthermore, it is desirable that more in vivo experiments proving favorable therapeutic indexes for the specific compounds become increasingly available. Nevertheless, we believe that certainly tricarbonyl complexes of rhenium, and possibly its dicarbonyl compounds, are poised to make an ever-increasing impact in the development on the new generation of metal-based antibiotic and anticancer drugs. Dicarbonyl compounds, in particular, should allow for even greater structural variations and design, owing to the extra coordination site available for ligand binding. It is, however, necessary to further explore and study the chemistry of the *cis*-[Re(CO)_2_]^+^ complexes, as the same remains greatly underdeveloped compared to that of *fac*-[Re(CO)_3_]^+^ compounds.
